# Associations of blood lipids and LDL cholesterol lowering drug-targets with colorectal cancer risk: a Mendelian randomisation study

**DOI:** 10.1038/s41416-024-02900-7

**Published:** 2024-11-23

**Authors:** Wing Ching Chan, Lili Liu, Emmanouil Bouras, Verena Zuber, Wanqing Wen, Jirong Long, Dipender Gill, Neil Murphy, Marc J. Gunter, Themistocles L. Assimes, Luis Bujanda, Stephen B. Gruber, Sébastien Küry, Brigid M. Lynch, Conghui Qu, Minta Thomas, Emily White, Michael O. Woods, Ulrike Peters, Christopher I. Li, Andrew T. Chan, Hermann Brenner, Konstantinos K. Tsilidis, Wei Zheng

**Affiliations:** 1 Department of Epidemiology and Biostatistics, School of Public Health, Imperial College London, London, UK; 2 Division of Epidemiology, Department of Medicine, Vanderbilt Epidemiology Center, Vanderbilt-Ingram Cancer Center, Vanderbilt University Medical Center, Nashville, TN USA; 3 Department of Hygiene and Epidemiology, University of Ioannina School of Medicine, Ioannina, Greece; 4 MRC Centre for Environment and Health, School of Public Health, Imperial College London, London, UK; 5 UK Dementia Research Institute at Imperial College, Imperial College London, London, UK; 6 Nutrition and Metabolism Branch, International Agency for Research on Cancer, World Health Organization, Lyon, France; 7 VA Palo Alto Health Care System, Palo Alto, CA USA; 8 Department of Medicine, Division of Cardiovascular Medicine, Stanford University School of Medicine, Stanford, CA USA; 9 Department of Gastroenterology, Biodonostia Health Research Institute, Centro de Investigación Biomédica en Red de Enfermedades Hepáticas y Digestivas (CIBERehd), Universidad del País Vasco (UPV/EHU), San Sebastián, Spain; 10 Department of Medical Oncology & Therapeutics Research, City of Hope National Medical Center, Duarte, CA USA; 11 Nantes Université, CHU Nantes, Service de Génétique Médicale, Nantes, France; 12 Cancer Epidemiology Division, Cancer Council Victoria, Melbourne, VIC Australia; 13 Centre for Epidemiology and Biostatistics, Melbourne School of Population and Global Health, The University of Melbourne, Melbourne, VIC Australia; 14 Division of Public Health Sciences, Fred Hutchinson Cancer Research Center, Seattle, WA USA; 15 Department of Epidemiology, University of Washington, Seattle, WA USA; 16 Memorial University of Newfoundland, Discipline of Genetics, St. John’s, NL Canada; 17 Clinical and Translational Epidemiology Unit, Massachusetts General Hospital, Boston, MA USA; 18 Division of Clinical Epidemiology and Aging Research, German Cancer Research Center (DKFZ), Heidelberg, Germany; 19 Division of Preventive Oncology, German Cancer Research Center (DKFZ) and National Center for Tumor Diseases (NCT), Heidelberg, Germany; 20 German Cancer Consortium (DKTK), German Cancer Research Center (DKFZ), Heidelberg, Germany

**Keywords:** Cancer epidemiology, Colorectal cancer

## Abstract

**Background:**

Whether blood lipids are causally associated with colorectal cancer (CRC) risk remains unclear.

**Methods:**

Using two-sample Mendelian randomisation (MR), our study examined the associations of genetically-predicted blood concentrations of lipids and lipoproteins (primary: LDL-C, HDL-C, triglycerides, and total cholesterol), and genetically-proxied inhibition of HMGCR, NPC1L1, and PCSK9 (which mimic therapeutic effects of LDL-lowering drugs), with risks of CRC and its subsites. Genetic associations with lipids were obtained from the Global Lipids Genetics Consortium (*n* = 1,320,016), while genetic associations with CRC were obtained from the largest existing CRC consortium (*n* = 58,221 cases and 67,694 controls). Our main analysis was a multivariable MR (MVMR) with mutual adjustments for LDL-C, HDL-C, and triglycerides. Secondary analyses, including MVMR additionally-adjusting for BMI or diabetes, were also performed.

**Results:**

Genetically-predicted LDL-C was positively associated with CRC risk in the MVMR adjusted for HDL-C and triglycerides (OR = 1.09; 95%CI 1.02–1.16 per SD increase) and additionally-adjusted for BMI (OR = 1.12; 95%CI 1.05–1.21) or diabetes (OR = 1.09; 95%CI 1.02–1.17). Associations were generally consistent across anatomical subsites. No clear evidence of association was found for other lipids, lipoproteins, or LDL-lowering drug-targets.

**Conclusions:**

We found evidence of a weak positive association between LDL-C and CRC that did not appear to be explained by potential pleiotropic pathways such as via HDL-C, triglycerides, BMI, or diabetes.

## Introduction

In 2020, colorectal cancer (CRC) accounted for over 1.9 million new cancer cases and 0.9 million deaths globally, making it the third most commonly-diagnosed cancer and the second leading cause of cancer death [1]. Extensive research into the modifiable risk factors of CRC has identified obesity, physical inactivity, smoking, heavy alcohol use and processed meat consumption as likely risk factors, with particularly strong evidence for body fatness [2, 3]. Although the biological mechanisms linking these factors to the development of CRC are multi-fold, all of the above risk factors were found to influence blood lipid levels [4–7]. Obesity, in particular, is closely linked to hyperlipidaemia, whereby free cholesterol, triglycerides and other lipids from adipose tissues are released into the bloodstream leading to abnormal levels of circulating lipids [8, 9]. Understanding the potential causal relevance of lipids in the development of CRC, which is currently still inconclusive, may uncover mechanistic pathways for obesity and diet and opportunities for intervention. It is also valuable to explore whether lipids are causes of CRC independent of established lifestyle risk factors.

Mendelian randomisation (MR) has been increasingly adopted to assess causality as it circumvents several key limitations of observational studies such as reverse causation and residual confounding. Existing MR studies on the associations between blood lipids and CRC risk have reported mixed results, but most of these studies either had limited statistical power (particularly for specific lipid fractions) [10–14], did not examine associations by CRC subsites or for early-onset CRC (i.e., diagnosed in <50 years-old) [11, 13–16], or only conducted univariable MR analysis which does not account for the pleiotropic nature of lipids-associated variants [11, 15, 16]. Specifically, as low-density lipoprotein cholesterol (LDL-C), high-density lipoprotein cholesterol (HDL-C) and triglycerides are highly-correlated and have shared genetic variants, analysis using a multivariable MR (MVMR) approach, which incorporates the genetic associations for several exposures into one model, could provide insight into the possible independent association of each lipid with CRC risk [17–19]. In addition, the relationship between LDL-lowering drugs such as statins and CRC risk has been explored in a small number of MR and observational studies, but findings are still inconclusive [11, 14, 20–23].

As such, the current study aimed to examine the associations of genetically predicted circulating lipid and lipoprotein concentrations with the risks of CRC, its anatomical subsites, and early-onset CRC. In particular, we were interested in assessing the independent effect of LDL-C, HDL-C, and triglycerides. We also evaluated the associations of total cholesterol, apolipoprotein A (ApoA), apolipoprotein B (ApoB), and lipoprotein A (Lp(a)). We then examined the associations of CRC with variants in the genes encoding 3-Hydroxy-3-methylglutaryl coenzyme A reductase (HMGCR), Niemann-Pick C1-Like 1 (NPC1L1), and proprotein convertase subtilisin/kexin type 9 (PCSK9), which are the targets of three commonly used LDL-lowering drugs (statins, ezetimibe, and PCSK9 inhibitors, respectively).

## Materials and Methods

**Fig. 1:**
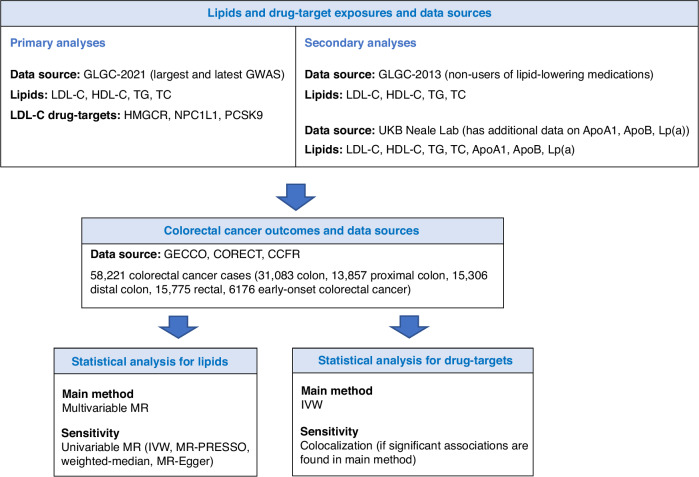
Study overview. GLGC Global Lipids Genetics Consortium, LDL-C low-density lipoprotein cholesterol, HDL-C high-density lipoprotein cholesterol, TG triglycerides, TC total cholesterol, HMGCR 3-Hydroxy-3-methylglutaryl coenzyme A reductase, NPC1L1 Niemann-Pick C1-Like 1, PCSK9 proprotein convertase subtilisin/kexin type 9, GECCO Genetics and Epidemiology of Colorectal Cancer Consortium, CORECT Colorectal Transdisciplinary Study, CCFR Colon Cancer Family Registry, IVW inverse-variance weighted.

An overview of our study is presented in Fig. 1. Our study is reported following the STROBE-MR (*“Strengthening the Reporting of Observational Studies in Epidemiology using Mendelian Randomisation”*) guidelines (Additional file 1).

### Study design

We adopted a two-sample MR approach [24], with data on the gene-exposure association obtained from the Global Lipids Genetics Consortium (GLGC) and the UK Biobank (UKB), and data on the gene-outcome association obtained from the Genetics and Epidemiology of Colorectal Cancer Consortium (GECCO), the Colon Cancer Family Registry (CCFR), and the Colorectal Transdisciplinary Study (CORECT), as described below.

### Data sources for exposures (lipids and LDL-C drug-targets)

For our main analyses, summary-level data on lipids (LDL-C, HDL-C, log-triglycerides, and total cholesterol) and drug-targets were obtained from the 2021 GWAS meta-analysis conducted by GLGC [25]. In this GLGC GWAS meta-analysis, data from five genetic ancestries were collected and both ancestry-specific and multi-ancestry meta-analyses were conducted. Only data from the European ancestry-specific analyses (which involved 1.3 million participants from 146 cohorts) were used in our present study as our outcome dataset was of European ancestry. In each included cohort, sex-specific residuals were generated with adjustments for age, age-squared, principal components (PCs) of ancestry, and cohort-specific covariates, and the residuals were then transformed using inverse-normalisation. In addition, the LDL-C and total cholesterol values for participants on lipid-lowering medications were divided by 0.7 and 0.8, respectively, as an estimation of their “pre-medication” values [25, 26]. Further details of the GLGC GWAS meta-analysis, including the genotyping and quality control (QC) procedures, can be found in the publication by Graham et al. [25]

For our secondary analyses, summary data on lipids and lipoproteins (LDL-C, HDL-C, triglycerides, total cholesterol, ApoA, ApoB and Lp(a)) were obtained from the second round of GWAS conducted by Neale et al. amongst UKB participants [27, 28]. Although the 2021 GLGC GWAS meta-analysis included samples from the UKB, the UKB GWAS by Neale et al. provided additional information such as sex-specific summary estimates and results on ApoA, ApoB and Lp(a), which allows us to have a more thorough investigation of the lipid-CRC association. Details of the sample collection, genotyping, and QC procedures in the UKB have been described elsewhere [29, 30]. The sex-combined GWAS by Neale et al. included 361,194 individuals of white-British ancestry and associations were adjusted for age, age-squared, sex, age×sex and age-squared×sex, and the first 20 PCs. The sex-specific GWASs, which included 194,174 females and 167,020 males, were adjusted for age, age-squared and the first 20 PCs [27, 28]. We also performed additional analyses using data from an earlier (2013) GWAS meta-analysis by GLGC that excluded individuals on lipid-lowering medications (*n* = 188,577) [31]. For consistency across lipids and datasets, inverse-rank normalised data were used.

### Data sources for outcomes

Summary-level data for the associations of genetic variants with risks of CRC and cancers of the colon, proximal colon, distal colon, and rectum were obtained from the latest GWAS meta-analysis involving 45 studies from GECCO, CORECT and CCFR [32]. Details on the genotyping, imputation, and QC procedures have been published by Huyghe et al. [32] In total, 58,221 CRC cases (of which 31,083 were colon, 13,857 were proximal colon, 15,306 were distal colon, 15,775 were rectal, and 6176 were early-onset CRC) and 67,694 controls primarily of European ancestry were included in the GWAS meta-analysis [32–34]. Sex-specific GWAS results were also available in 66,026 men and 59,889 women [33, 34]. Associations with CRC (and subsites) were obtained from logistic regression models that adjusted for age, sex, study, and PCs, and estimates from individual studies were then combined using fixed-effect inverse-variance weighted meta-analyses [32].

### Statistical analyses

#### Part 1. Lipids and CRC

As previously mentioned, many lipids are highly-correlated, with shared genetic variants. Therefore, when studying the potential effect of individual lipids, the “exclusion restriction” assumption (i.e., the IVs influence the outcome only via the exposure) may not hold and there may be horizontal pleiotropy between lipids. Multivariable MR (MVMR) can “mitigate horizontal pleiotropy via known pleiotropic pathways through the inclusion of multiple exposures” and enables the assessment of the independent association of each included lipid with CRC risk [17, 19, 35]. As such, MVMR was chosen as our main analysis approach, with LDL-C, HDL-C and triglycerides included in the same model [17–19]. In selecting the genetic instruments for MVMR, a SNP was considered eligible for inclusion if it was significantly associated (at *p* < 5×10^-8^) with at least one of the lipids included in the model. For these SNPs, the summary data from the lipids-GWAS and CRC-GWAS were then combined, and “inconsistent” SNPs whose effect allele (in one dataset) matched with neither the effect nor non-effect allele (in the other dataset) were excluded. Data harmonisation was then conducted by aligning the effect allele (of each SNP) from the lipids and CRC datasets, such that the estimates from the SNP-lipids and SNP-cancer associations correspond to the same allele. To remove SNPs that are in high linkage-disequilibrium (LD) with each other, SNPs were then clumped based on an r^2^ threshold of <0.001 and a clumping distance of 10,000 kb. The remaining SNPs comprised the genetic instruments for our lipids MVMR analyses.

To account for other potential horizontal pleiotropic pathways, we checked whether our IVs were significantly associated with established CRC risk factors that have been suggested to be linked to lipids, using the PhenoScanner database (http://www.phenoscanner.medschl.cam.ac.uk/). BMI and Type 2 diabetes were found to be frequently associated with our IVs, and since there is no compelling evidence that they mediate the lipids-CRC associations, they were additionally included into the MVMR model as sensitivity analyses to assess possible horizontal pleiotropy.

Total cholesterol was investigated using univariable MR (UVMR) as it could not be included in the MVMR model with the other three lipid fractions that make up total cholesterol. For comparison purposes, we also conducted UVMR as sensitivity analyses for LDL-C, HDL-C, and triglycerides. We used the random-effects inverse-variance weighted (IVW) method, as well as univariable approaches that are more robust to influences from potential horizontal pleiotropy (i.e., weighted median, MR-Egger and MR-PRESSO) [24, 36–39]. Findings from these robust MR methods were compared with our MVMR results. To test the “relevance” assumption (i.e., the genetic IVs are significantly associated with the exposure of interest), we calculated the proportion of variance in each lipid explained by its corresponding IVs and the F-statistics of these IVs (which reflect instrument strength, with a value of >10 typically used as evidence against weak instrument bias) [24, 35]. Specifically, for MVMR, the conditional F-statistics were calculated to assess whether the IVs “strongly predict each exposure conditional on the other exposures included in (the) MVMR model” [35].

For our secondary exposures (ApoA, ApoB and Lp(a)), we conducted MVMR with Lp(a), LDL-C, HDL-C, and triglycerides included in the same model, but ApoA and ApoB were not included to prevent multicollinearity as they are too highly-correlated with HDL-C (r = 0.98) and LDL-C (r = 0.98), respectively. Instead, they were only explored using UVMR. Using the UKB sex-specific lipids-GWAS, we also performed MVMR analyses in men and women separately and tested for potential heterogeneity by sex. MVMR and UVMR analyses were conducted using the R packages “*MendelianRandomization”* and/or “*MRPRESSO”*.

#### Part 2. LDL-C drug-targets and CRC

Using the 2021 GLGC lipids data, SNPs that were associated with LDL-C at the genome-wide significance threshold of *p* < 5 × 10^-8^, and within 100 kb from the *HMGCR* (Entrez Gene: 3156), *NPC1L1* (Entrez Gene: 29881), and *PCSK9* (Entrez Gene: 255738) genes, respectively, were selected [40]. Effect alleles were oriented to correspond to a reduction in LDL-C. These SNPs were then clumped at an r^2^ of <0.2 and a clumping distance of 250 kb, and the resulting SNPs were used as genetic IVs to proxy the inhibition of HMGCR, NPC1L1, and PCSK9. The summary data from the LDL-GWAS and CRC-GWAS were then combined for these SNPs and effect alleles in the exposure and outcome datasets were harmonised. The associations of genetically-proxied inhibition of HMGCR, NPC1L1, and PCSK9 with CRC risk were investigated using the IVW method, adjusting for weak correlations between SNPs (calculated with reference to the 1000 Genomes panel), with estimates scaled to reflect the odds of CRC associated with 1-SD reduction in LDL-C. We conducted sensitivity analyses using independent (clumped using an r^2^ of <0.001) rather than weakly-correlated (r^2^ < 0.2) SNPs, as well as a positive control analysis with coronary artery disease (CAD) as the outcome to test the validity of our instruments [41].

Where significant associations with CRC or subsites were identified, colocalization analyses were performed using the *Coloc* R package, which applies a Bayesian framework to determine whether there are causal variants shared between two traits at a specified gene region and provides insight as to whether our observed MR associations were due to genetic confounding by high LD between SNPs [42, 43]. Posterior probabilities (PP) were calculated for testing the following hypotheses: no causal variant for drug-targets or CRC (H0); causal variant for drug-targets only (H1) or CRC only (H2); distinct causal variants for drug-targets and CRC (H3); and shared causal variant for the two traits (H4) [42, 43]. If the PP under H4 was >0.5, or if PP(H4) > PP(H3) and PP(H4) + PP(H3) > 0.5, then we would conclude that our drug-target MR results were not likely to be explained by genetic confounding [42, 43].

## Results

### Part 1. Lipids and CRC

Summary information of the included IVs for each lipid, and their associations with the exposures and outcomes, are presented in Additional file 2: Tables S1-S4. The estimated power to detect an OR of 1.10 per SD increase in our primary exposures (LDL-C, HDL-C, triglycerides, total cholesterol) was >80% for CRC, colon, and rectal cancer (Additional file 2**:** Table S1). The proportion of variance explained ranged from 6.0 to 7.6%, while the F-statistics ranged from 268 to 338 (conditional F-statistics: 68 to 175), indicating no clear evidence of weak instrument bias [24, 35].

#### Total cholesterol

Genetically predicted total cholesterol was not associated with overall CRC (OR_IVW_ = 1.05; 95%CI: 0.99–1.12 per SD increase), but there was some evidence of positive associations with colon, particularly distal colon, cancer, with OR_IVW_ of 1.07 (1.00–1.15) and 1.14 (1.05–1.24), respectively (Additional file 2: Tables S6).

#### LDL-C

**Fig. 2:**
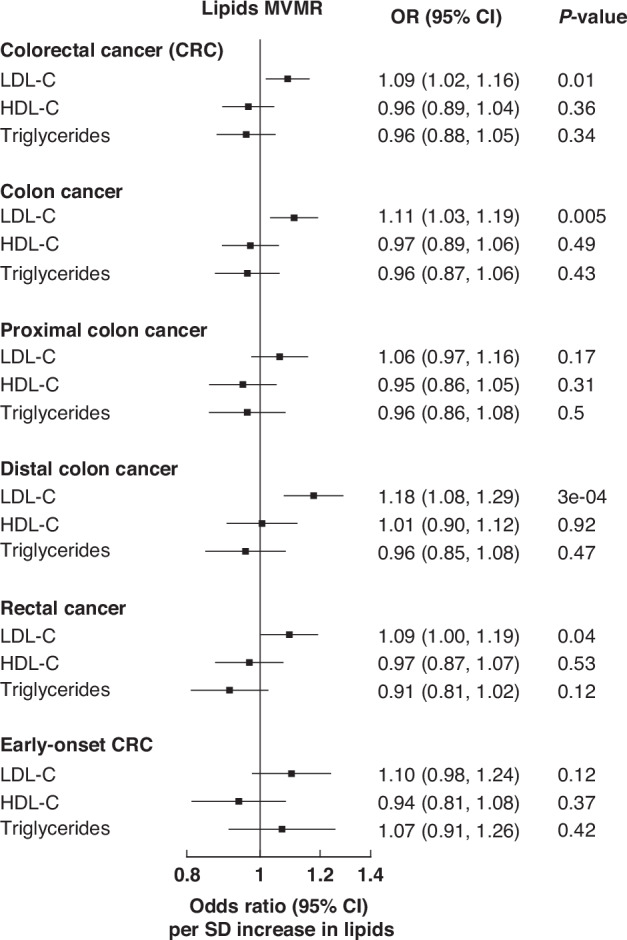
Multivariable MR (MVMR) estimates of the associations between lipids and colorectal cancer (overall and by subsites). Odds ratios were obtained from multivariable IVW models that included the genetic associations of LDL-C, HDL-C, and triglycerides. The odds ratio for each lipid therefore represents its association with the outcome after accounting for the effects of the other two lipids. Error bars represent 95%CIs. 492 SNPs were used in this MVMR analysis. Conditional F-statistics for LDL-C, HDL-C, and triglycerides were 68, 175, and 156, respectively. OR Odds Ratio, CI confidence intervals, LDL-C low-density lipoprotein cholesterol, HDL-C high-density lipoprotein cholesterol, SD standard deviation.

Using the 2021 GLGC lipids data, our MVMR analyses showed that genetically predicted LDL-C was positively associated with CRC risk (OR = 1.09; 95%CI: 1.02–1.16 per SD increase in LDL-C) after mutually-adjusting for HDL-C and triglycerides (Fig. 2). Associations were generally consistent across CRC subsites; LDL-C appeared to be slightly more strongly associated with distal colon cancer (1.18; 1.08–1.29) than with proximal colon cancer (1.06; 0.97–1.16; *p*-*heterogeneity* = 0.1). Compared with overall CRC, no stronger association with LDL-C was found for early-onset CRC (1.10; 0.98–1.24; *p*-*heterogeneity* = 0.9) but power was limited for the latter outcome. Associations were broadly similar when the genetic effects of BMI or diabetes were also accounted for in the MVMR models (Additional file 2**:** Tables S7 and S8).

Genetically predicted LDL-C was also positively associated with CRC risk in our univariable IVW analysis (1.08; 1.01–1.16 per SD increase); although the association was attenuated in the weighted-median, MR-Egger and MR-PRESSO analyses, the direction of association remained consistent (Additional file 2: Table S6).

In our secondary analyses using other lipids-GWAS, genetically predicted LDL-C was also positively associated with CRC risk in UKB (OR_MVMR_ = 1.10; 1.02–1.19 per SD increase; Additional file 2**:** Tables S9 and S10), but not in 2013-GLGC although the direction of association was consistent (OR_MVMR_ = 1.05; 0.99–1.12; Additional file 2**:** Table S11). There were no clear sex-differences for the associations between LDL-C and CRC (and its subsites) (*p-heterogeneity* > 0.05; Additional file 2**:** Table S9).

#### HDL-C, triglycerides, and other lipoproteins

Genetically predicted HDL-C and triglycerides were not associated with the risks of CRC or its subsites in the MVMR model (OR = 0.96; 0.89–1.04 and 0.96; 0.88–1.05 per SD increase in HDL-C and triglycerides, respectively; Fig. 2). Similar associations were observed in the MVMR additionally accounting for BMI or diabetes (Additional file 2**:** Tables S7 and S8), in the UVMR analyses (Additional file 2**:** Table S6), and when the UKB or 2013-GLGC lipids-GWAS were used (Additional file 2**:** Table S9–11). No clear sex-differences were found for the associations between these lipids and CRC (and its subsites) (*p-heterogeneity* > 0.05) (Additional file 2**:** Table S9).

Our investigation of other lipoproteins using the UKB lipids-GWAS showed no clear association between Lp(a) and CRC after adjusting for LDL-C, HDL-C, and triglycerides (OR_MVMR_ = 1.03; 0.96–1.11 per SD increase; Additional file 2**:** Table S9). There was a suggestive positive association of genetically predicted ApoB with CRC risk in the UVMR analyses (*p* = 0.09; Additional file 2**:** Table S10).

### Part 2. Drug-targets and CRC

**Fig. 3:**
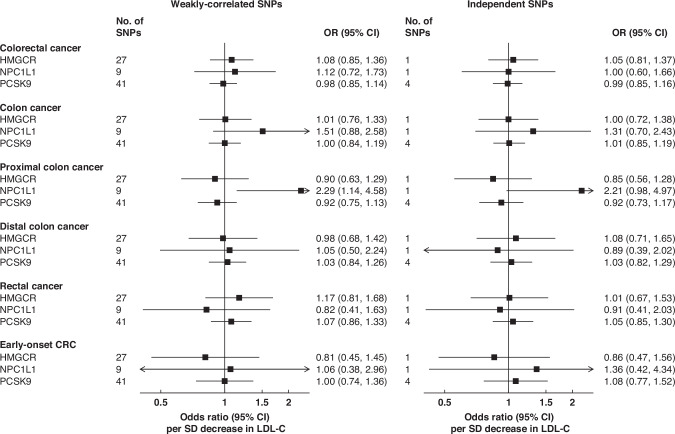
Associations of genetically-proxied inhibition of HMGCR, NPC1L1, and PCSK9 with colorectal cancer outcomes. The estimates are the odds ratios associated with a 1-SD reduction in LDL-C, with error bars representing 95%CIs. Associations on the left panel were adjusted for the correlations between SNPs. OR Odds Ratio, CI confidence intervals, LDL-C low-density lipoprotein cholesterol, SD standard deviation.

Overall, 27, 9 and 41 SNPs were included as genetic IVs for the inhibition of HMGCR, NPC1L1 and PCSK9, respectively (F > 10 for each included SNP, see Additional file 2**:** Tables S1 and S5). The statistical power for the drug-target analyses were low (10-66% to detect an OR of 1.10; Additional file 2**:** Table S1). Most of the LDL-lowering drug-targets examined were not associated with CRC or its subsites, except for genetically-proxied NPC1L1 inhibition which was associated with higher proximal colon cancer risk (OR = 2.29; 1.14-4.58 per SD decrease in LDL-C), but this association was no longer statistically-significant when independent SNPs were used (Fig. 3) and was not supported by our colocalization results (Additional file 2**:** Table S12**)**.

In our positive control analyses, genetically-proxied inhibition of HMGCR, NPC1L1 and PCSK9 were all significantly associated with lower CAD risk (corresponding ORs = 0.63; 0.50–0.80, 0.54; 0.33–0.88, and 0.51; 0.43–0.60 per SD decrease in LDL-C), consistent with existing knowledge.

## Discussion

Using a two-sample MR approach, our study investigated the associations of lipid and lipoprotein concentrations and LDL-lowering drug-targets with the risks of CRC and its anatomical subsites. Our main findings were the weak positive associations between genetically predicted LDL-C and the risks of CRC, colon, distal colon, and rectal cancer, after accounting for the potential influence of other lipids. Associations in our sensitivity analyses were directionally-concordant (although some were non-significant) with our main results. No significant associations were observed for genetically predicted HDL-C, triglycerides, or other lipoproteins. We also found no clear evidence of associations between genetically-proxied inhibition of HMGCR, NPC1L1, or PCSK9 and risks of CRC and its subsites.

A recent meta-analysis that included 15 prospective observational studies on total cholesterol and CRC (20,454 cases) reported a significant positive association, with a summary RR of 1.15 (1.08–1.22) when comparing the highest vs. lowest cholesterol category [44]. Similarly, three MR studies also found significant positive associations between genetically predicted total cholesterol and CRC prior to correction for multiple-testing [11, 15, 16]. In our study, total cholesterol was only positively associated with colon (particularly distal colon) cancer, and some of its associations were similar to the UVMR associations observed for LDL-C, suggesting that the positive associations found for total cholesterol may be driven by LDL-C. However, as total cholesterol is a heterogeneous exposure that includes several lipid fractions with different putative mechanisms, it is important to disentangle the effects of individual lipids using MVMR.

To our knowledge, only the MR studies by Cornish et al. and Bull et al. had adequate power to detect small to moderate associations with specific lipid fractions [15, 33], which may partly explain the non-significant associations observed in most previous MR studies [11–13, 16]. For LDL-C, the significant positive association with CRC found in our study is consistent with the finding from the two-sample MR study by Cornish et al. (OR = 1.14; 95%CI 1.04–1.25 per SD increase in genetically predicted LDL-C), which included 26,397 CRC cases but only performed UVMR analyses [15]. The two-sample MR by Bull et al. (with ~58,000 CRC cases and genetic associations of lipids obtained from a GWAS of nuclear magnetic resonance metabolomics [*n* = 13,000 to 25,000]) found an association of LDL-C with only distal colon cancer, but they similarly did not account for HDL-C and triglycerides, as the aim of their study was to investigate mediation by metabolites in the adiposity–CRC association [33]. Two previous MR studies have examined multiple lipid fractions simultaneously using a MVMR approach [12, 14], with one study utilising CRC GWAS data from FinnGen (843 cases) and the other data from UKB (5486 cases). The latter study reported a significant positive association between genetically predicted LDL-C and CRC risk after accounting for genetically predicted HDL-C and triglycerides (OR = 1.16; 95% CI 1.04–1.29 per SD increase) [14]. With over 10-times the number of CRC cases, our study supported this finding and additionally showed that the positive association with LDL-C was not likely explained by possible pleiotropic effects from BMI or diabetes. Our study was also one of the few that investigated CRC subsites, and we found that the significant positive association with LDL-C was consistently observed in most subsites.

Despite our observation of positive associations with genetically predicted LDL-C, LDL-lowering drug-targets were generally not significantly associated with the outcomes studied. This could be due to the low statistical power and proportion of variance explained for each drug-target, particularly since the associations between LDL-C and CRC endpoints were relatively weak. Previous MR studies on genetically-proxied inhibition of HMGCR, NPC1L1 and PCSK9, as well as RCTs and prospective cohort studies on the corresponding drugs, have yielded mixed findings. For example, genetically-proxied inhibition of HMGCR, which mimics the effect of statins, was found to be associated with a lower risk of CRC (OR = 0.69; 95%CI 0.49–0.99) in the study by Rodriguez-Broadbent et al. that involved a 6-SNPs genetic risk score and 9254 CRC cases [11], but not in the study by Carter et al. using the same 6-SNPs and 5486 cases from the UKB (1.05; 0.65-1.72 per SD decrease in LDL-C) [14]. Meta-analyses of RCTs with CRC as a secondary outcome have generally found no association with statin use, but most had relatively short follow-up periods (<5 years) and a small number of events [23, 45, 46]. A 2014 meta-analysis of 13 prospective cohort studies reported a significant inverse association between statin use and CRC risk (RR = 0.93; 0.87–0.99) [23], but there was substantial heterogeneity between studies, and potential residual confounding (for example, from lifestyle changes accompanying statin use) may have influenced the association.

As for genetically-proxied inhibition of NPC1L1, which mimics the effect of ezetimibe, although we found a suggestive association with proximal colon cancer risk, this was not supported by colocalization and could be due to potential genetic confounding or may be a chance finding. In other similar MR studies, genetically-proxied inhibition of NPC1L1 was associated with higher CRC risk in the two-sample MR by Carter et al. (OR = 2.13; 1.18–3.85 per SD decrease in LDL-C; 5 SNPs as IVs) [14], but not with colon cancer risk in a one-sample MR study from Denmark that utilised a 4-SNPs risk score and included 1144 cases [21]. A 2022 meta-analysis of four RCTs found ezetimibe use to be associated with an increased risk of intestinal cancer (small and large intestine combined), but there were only ~100 cases in each treatment arm [47]. For PCSK9, no significant association with CRC was found in the only other MR study identified (797 events, 0.40; 0.14-1.19 per 1 mmol/L decrease in LDL-C) [20], and no relevant RCT or meta-analysis of cohort studies was identified.

Lastly, the associations of HDL-C and triglycerides with CRC risk are still unclear. Although the latest meta-analysis of prospective cohort studies reported a significant inverse association with HDL-C (5,869 cases from 11 studies; summary RR = 0.86; 0.77–0.97 for highest vs. lowest category) and a significant positive association with triglycerides (11,023 cases from 13 studies; summary RR = 1.21; 1.09–1.34) [44], previous MR studies have all reported no significant associations with these lipids and our findings provided further support to this [11–16].

To our knowledge, the current study is the largest MVMR study of blood lipids and CRC to date. With over 58,000 CRC cases, we have ≥80% power to detect (or refute) small-to-moderate associations, particularly for LDL-C, HDL-C, and triglycerides, which most previous MR studies did not have sufficient power for. Moreover, we have used lipids data from the latest (2021) GLGC GWAS meta-analysis in our primary analyses (as opposed to the 2013 GLGC meta-analysis that was used in most previous MR studies), which included a larger number of studies and samples and allowed more genetic variants to be identified, thus improving the proportion of variance explained and statistical power. Another strength of our study is the use of MVMR to study the effect of multiple lipids simultaneously whilst accounting for each other, as well as to account for possible pleiotropy from BMI or diabetes. This is particularly important in the case of lipids as they are highly-correlated with each other, and MVMR allows us to attempt to disentangle their effects. Finally, our study is one of the few MR studies to examine the relationships between lipids and subsites of CRC and to explore potential heterogeneity by sex, but despite being the largest study to date, power was still limited for some of the subsites.

There are several limitations in our study. Firstly, the associations with genetically predicted LDL-C were relatively weak in magnitude and could theoretically be driven away by small biases. Secondly, limited power was an issue for our drug-target analyses and for early-onset CRC. As such, larger power is needed to provide final conclusions. Thirdly, our analyses were conducted in individuals of European ancestry; as such, our results may not be generalisable to other ethnic groups. Fourth, there was a small proportion (<5%) of sample overlap between our exposure and outcome samples, but simulation studies have demonstrated that the bias associated with a small overlap (and no weak instruments) is likely negligible [48]. Lastly, it was not possible to explore potential non-linear associations using summary data but there is no compelling evidence of non-linear associations between lipids and CRC in existing observational studies.

## Conclusions

In conclusion, our study found evidence of a weak positive association between LDL-C and CRC that did not appear to be due to alternate pathways such as via HDL-C, triglycerides, BMI, or diabetes. We did not observe any associations for HDL-C, triglycerides, or LDL-lowering drug-targets. Given the weak magnitude of association with LDL-C and the lack of evidence of associations with the LDL-lowering drug-targets, future larger studies are warranted to provide more definitive conclusions.

## Supplementary information

Additional File 1. STROBE-MR checklist

Additional File 2. Supplementary Tables

Additional File 3. Funding and Acknowledgements for GECCO

## Data Availability

The summary-level GWAS statistics for the exposures were obtained from the Global Lipids Genetics Consortium [25, 31] and from Neale et al. (https://www.nealelab.is/uk-biobank) [27, 28]. The summary-level GWAS statistics for the outcomes are available via application to the Genetics and Epidemiology of Colorectal Cancer Consortium (GECCO). Code may be made available upon reasonable request to the study investigators.
